# How Fast Is the Sessile Ciona?

**DOI:** 10.1155/2009/875901

**Published:** 2009-12-16

**Authors:** Luisa Berná, Fernando Alvarez-Valin, Giuseppe D'Onofrio

**Affiliations:** ^1^Laboratory of Animal Physiology and Evolution, Stazione Zoologica Anton Dohrn, 80121 Napoli, Italy; ^2^Sección Biomatemática, Facultad de Ciencias, Universidad de la República, Igua 4225, 11400 Montevideo, Uruguay

## Abstract

Genomewide analyses of distances between orthologous gene pairs from the ascidian species *Ciona intestinalis* and *Ciona savignyi* were compared with those of vertebrates. Combining this data with a detailed and careful use of vertebrate fossil records, we estimated the time of divergence between the two ascidians nearly 180 My. This estimation was obtained after correcting for the different substitution rates found comparing several groups of chordates; indeed we determine here that on average Ciona species evolve 50% faster than vertebrates.

## 1. Introduction

The ascidian (sea squirts) species of the genus Ciona are one of the most prevalent modern urochordates or tunicates. These organisms, along with cephalochordates, occupy a key evolutionary position. For this reason they have been considered fundamental in order to clarify the origins of chordates, the origins of vertebrates from simpler organisms, as well as the mechanisms of vertebrate development.

The most widely studied Ciona species are *C. intestinalis* and *C. savignyi*. The former is cosmopolitan while the latter is geographically restricted to the northern Pacific coast. The two species are very similar in their morphology, and indeed, cases of misidentification have been reported [1, and references therein]. In fact, the adult of the two species can only be distinguished by minor features such as the presence of an endostylar appendage in *C. intestinalis*, absent in *C. savignyi*, and by the location of the pharingeo-epicardic openings. Although no hybrids of the two species have been recognized in nature, they can be experimentally obtained by fertilizing either species' dechorionated eggs with heterologous sperm from the other. These hybrids are able to develop up to the tadpole stage [2]. 

While morphology and the possibility of producing hybrids suggest that these two Ciona species are close relatives, the comparisons using sequence data indicate that they are rather divergent [3]. In regard to the above, it is worth mentioning that even inside the *C. intestinalis* species group, the divergence is quite remarkable [4]. The distance between two cryptic species of *C. intestinalis* has been estimated using 1051 aligned amino acids obtained by the concatenation of 13 genes [5–7]. According to this study, calibrating the molecular clock by invertebrate and vertebrate fossil records, the time of divergence would be between 28 and 37 My. Using the same data, we estimated the time of divergence between *C. intestinalis* and *C. savignyi* in the range of 200–280 My (the lower and higher estimates are based on insect and vertebrate molecular clocks, resp.). However, the same authors [5–7] pointed out that the data reported about the time of divergence were partially influenced by several factors affecting the precision of the estimation. More specifically, the speed of molecular evolution of Ciona species is not known and besides its relative rate in comparison to the species used as reference was not assessed [5]. On the other hand, comparative analysis of the huntingtin gene from Ciona and several vertebrates indicates that the two Ciona species have a “protein divergence comparable to that observed between mammals and fishes,” concluding that they have either “an ancient origin” or “a high evolutionary rate” [8]. Similar conclusions were reached using 18S rRNA sequences [3]. According to this work, a great nucleotide divergence between *C. intestinalis* and *C. savignyi* was observed and the estimated distance was less than that between human and frog but greater than that observed between human and chicken [3].

Given that the draft genome sequences from *C. intestinalis* and *C. savignyi *are available since 2002 and 2005, respectively, [7, 9], it is now possible to make more accurate assessment of sequences divergence (with low impact of stochastic effects due to the fact that we use large gene samples). In addition, the genomes of two deuterostomes were published recently, namely, those of sea urchin [10] and amphioxus [11]. This situation is an excellent opportunity that allows us to perform whole-genome analysis in order to have accurate estimations of sequence distances. Since these two organisms are suitable outgroups permits to assess the relative speed of genome evolution in ascidians in comparison to vertebrates.

In summary, in the present work do not only we use a whole genome approach to obtain much more accurate estimates of distances between the two ascidians but also we combined this data with a detailed and careful use of fossil records taking into account different rates of evolution of vertebrates and ascidians.

## 2. Materials and Methods

### 2.1. Sequence and Fossil Record Data

Coding sequences of *C. intestinalis* were retrieved from the databases Ensemble (http://www.ensembl.org/) and JGI (http://genome.jgi-psf.org/), and for *C. savignyi*, *Monodelphis domestica*, and *Ornithorhynchus anatinus* from Ensemble. The genomic sequences of *Mus musculus*, *Rattus norvegicus*, *Bos taurus*, *Homo sapiens*, *Strongylocentrotus purpuratus,* and *Gallus gallus* were retrieved from NCBI (http://www.ncbi.nml.hih.gov/), while those of *Xenopus laevis* and *Branchiostoma florida* from http://www.xenbase.org/ and JGI, respectively. Orthologous gene pairs were identified by a Perl script, performing reciprocal Blastp [12] and selecting the Best reciprocal hit (BRH). The times of divergence in vertebrates, estimated from the fossil records, were collected from literature [13].

### 2.2. Sequence Alignment and Distance Calculation

Pairs of orthologous sequences were aligned by Clustal W [14]. The program Fprotdist with the Jones-Taylor-Thornton model (JTT) was used to calculate the amino acid distances [15]. In order to avoid bias distance estimations, due to a substitution saturation effect, pairwise distances >1 were disregarded for further analysis.

### 2.3. Relative Rate Test

To determine whether the same molecular clock applies to all chordates (namely ciona, amphioxus, and vertebrates), that is to say, if all chordates evolve at the same rate at the molecular level, the Tajima's relative rate test was conducted [16]. 

A relative rate test compares the relative substitution rates between two species (since their common ancestor) using as a reference a third species (outgroup) branching off earlier than the species to be tested. In particular, Tajima's test compares the number of substitutions between species 1 and outgroup (N13) with the number of substitutions between species 2 and outgroup (N23). The comparison is restricted to those sites in which both sequences 1 and 2 are different. The null hypothesis is that N13 = N23. The statistical significance of the difference between these two sequences is assessed by the chi-square (*χ* ^2^) test with one degree of freedom [16]. For the purpose of comparing ascidians and vertebrates substitution rate, orthologous genes shared by *C. intestinalis*, one vertebrate and an outgroup (amphioxus or sea urchin) were obtained with the same procedure described in the previous section. The three orthologous sequences were aligned using Clustalw [14], and a Perl script was developed to conduct the Tajima's test (Lamolle http://www.pasteur.edu.uy/blogs/anotacion/escripts/). This procedure was performed for each one of the most representative vertebrates for which their genomes are available (human, mouse, frog, opossum, platypus, cow, chicken, and fish).

### 2.4. Estimation of Acceleration Rate

In order to calculate the rate of acceleration of *C. intestinalis* versus a given vertebrate, their respective distances to a common ancestor (**a** and **b**) were determined [17, 18]. Let: (i) **d**_12_ = **a** + **b** the distance between *C. intestinalis* and the vertebrate to be tested; (ii) **d**_13_ = **a** + **D**1 + **D**2 be the distance between *C. intestinalis* and the outgroup; (iii) let **d**_23_ = **b** + **D**1 + **D**2 the distance between the vertebrate to be tested and the outgroup (see Figure 1S in Supplementary Materials available online at doi:10.1155/2009/875901). The rate of acceleration, using amphioxus or sea urchin as the outgroup, was estimated by (**a** − **b**)/**b**. Branch lengths were determined using the program Fprotdist, with the Jones-Taylor-Thornton model (JTT) [15].

## 3. Results and Discussion

Genomewide interspecies distances among several vertebrate species representative of different classes, as well as between the two Ciona species, were analyzed. To this aim, the first step was to identify orthologous gene pairs and then to calculate the amino acid distances. Nine different couples of vertebrates, mainly human and another species representative of different vertebrate classes (mouse, opossum, ornithorhynchus, cow, chicken, frog, and fugu), were analyzed. The orthologous gene pairs of *X. laevis* and *X. tropicalis *as well as *M. musculus* and* R. norvergicus* were also analyzed. The average amino acid distances (and their standard errors) between pairs of vertebrates and the two Ciona species, the number of orthologous pairs used for each comparison, and the time estimations obtained from fossil records [13] were reported in Table 1.

The average distance between the two Ciona species turned out to be higher than that between *H. sapiens* and *G. gallus,* but, lower than that between *H. sapiens* and *X. laevis.* In both cases the differences were statistically significant (Mann-Whitney nonparametric test, *P*-value <1.16e^−10^ and <2.2e^−16^ resp.). The results were in accordance with those reported by Johnson and coworkers [3], obtained with the 18S rRNA sequences.

**Figure 1:**
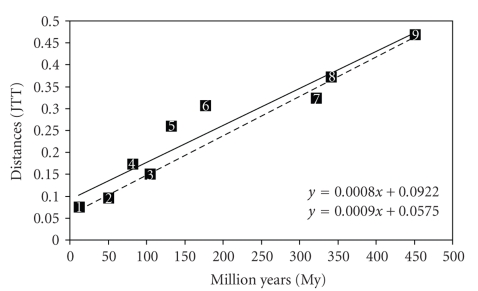
Regression line of divergence times derived from the fossil record (Table 1) and the correspondent distances (JTT method) in different vertebrate pairs. For the continuous line: 1. *M. musculus – R. norvergicus*, 2. *X. laevis – X. tropicalis*, 3. *H*. *sapiens – B*. *taurus*, 4. *H. sapiens – M. musculu*s, 5. *H. sapiens – M. domestica*, 6*. H. sapiens – O. anatinus*, 7*. H. sapiens – G. gallus, *8. *H. sapiens – X. laevis*, 9. *H. sapiens – T. rubripes. *For the dashed line: same pairs, comparisons involving nonplacental mammals (points 5 and 6) were excluded.

The graphical representation of the relationship between genomic divergence and time of divergence, in vertebrates only, has been presented in Figure 1. A clear linear relationship (i.e., molecular clock) was found, in good agreement with the conclusion achieved by likelihood-based method [11].

Yet two aspects deserve to be pointed out. First, contrary to what would be expected, the regression line does not pass through the origin (time equal zero should correspond to zero distance). This can be attributed to the fact that some of the orthologous pairs could be, most probably, paralogous. Second, the distances between human and marsupials or platypus were higher than expected (points 5 and 6 in Figure 1). This was better observed removing nonplacental mammals from the analysis, and indeed, all points fitted almost perfectly the regression line (dashed line). In other words, the result soundly supported that the rate of molecular evolution has been accelerated in non-placental mammals, that is, both monotremes and marsupials.

Assuming that the genomes of ascidians have the same evolutionary pace as the remaining chordate, without considering non-placental mammals, the estimation of the diverging time between the Ciona species would be approximately 308 (±16) My. Considering the close morphology between these ascidians, this estimation is surprisingly higher than expected. Moreover, the Early Cambrian fossil discovered in China several years ago [19], namely, the aplousobranch tunicate, shows an overall morphological aspect very similar to that of living species. Thus, in more than 500 My, the overall morphological features of ascidians' species seems to be poorly affected. Nevertheless, it is worth bringing to mind that all data reported in the previous paragraph about the estimation of diverging time were obtained under the assumption that the same molecular clock governs Ciona and the remaining vertebrates. However, phylogenetic analyses of single [8] or large set of nuclear genes [8, 11, 20, 21], as well as mitochondrial ones [20], have been suggested that the ascidian's genomes evolve faster than vertebrate ones. Therefore, the assumption that ascidians and remaining chordates have the same evolutionary pace should be carefully reconsidered. Some inaccuracy could probably affect the reported determination based on, or, more precisely, that the time of divergence was very probably an overestimate. Therefore, a genomewide analysis aiming first to verify whether this hypothesis is correct, and second to determine the acceleration extend, if any, was carried out.

In order to verify if vertebrates and ascidians have the same molecular clock, Tajima's relative rate test was conducted. For this purpose, orthologous sequences shared by *C . intestinalis*, one vertebrate, and one outgroup were analyzed. As outgroup the cephalochordate amphioxus, whose genome has been recently published [11], was used. The choice was largely justified by the new phylogeny of chordate organisms, according to which urochordates, instead of cephalochordate, are the closest relative of vertebrates, as previously accepted [11, 21–23]. However, an unsolved controversy still runs about this specific phylogenetic item; therefore the same analysis was performed using as outgroup the sea urchin, whose status is not questioned [20, 24, 25]. 

Besides, the comparisons of ascidians with several vertebrates were performed, in order to make sure that detectable and significant differences, if any, between Ciona and vertebrates were not comparison-specific, but reflecting a more general aspect of chordate evolution. Species from main vertebrate classes with the exception of reptiles, due to the lack the genome data, were used. As clearly emerged from Tajima's test results (Table 2), for the great majority of the alignments, Ciona genes evolve faster than those of all vertebrate groups, including the non-placental mammals, which, as discussed above, were characterized by the faster evolutionary rate among vertebrates. Indeed, the proportions of genes that evolve fast in Ciona ranged from 67% to 83%, according to the different vertebrate groups. Furthermore, considering only those alignments that yielded a significant *χ* ^2^ value, the proportion of genes faster in Ciona increased up to ~85%. The results remained basically unchanged using sea urchin as outgroup (Table 2). Thus, the evolutionary rate of Ciona at molecular level turned out to be faster than that of all other vertebrate species analyzed in this work.

In order to be able to use the calibration of vertebrate molecular clock (Figure 1), it was unavoidable to determine the extent of the acceleration in Ciona. The simplest approach was to estimate the branch lengths separating both Ciona and vertebrates from their common antecessor [18]. The results showing the acceleration rates between *C. intestinalis* versus human, mouse, frog, cow, chicken, and fish, using amphioxus as outgroup, were reported in Table 3. The distance **a**, relative to *C. intestinalis*, was always greater than the distance **b**, relative to each vertebrate, by a factor of 1.50. That is, on the average, Ciona evolves 50% faster than all vertebrates, with the exception of *O. anatinus* and *M. domestica*.

With this information on one hand and the calibration of molecular clock on the other (see Figure 1), the divergence between *C. intestinalis* and *C. savignyi* was reestimated and found to took place ~184 (±15) My ago. This new estimation, considerably inferior than our previous one obtained without adjusting the relative molecular clocks, fitted better with the observed morphology of living tunicates and specimens of an Early Cambrian fossil tunicate [1, 19]. Moreover, the result was in good agreement with the data about the rate of heterozygosity in both *C. intestinalis* and* C. savignyi*, the former showing, on the average, 1.2% of nucleotides differing between chromosome pairs of a single individual [9], and the latter, even much more polymorphic, with, on the average, 4.5% of SNP heterozygosity [6, 7]. These high polymorphism rates had been related with the effect of large effective population size [6] and a high recombination ratio reported in *C. intestinalis* [26].

As far as the subject of evolutionary rates is concerned, the results presented in this work are in agreement with previous ones in that Ciona species would have higher evolutionary rate than vertebrates [5, 8, 27]. It is worth bringing to mind that most probably such a fast rate is not a peculiar feature of the sessile ascidians. Among tunicates, indeed, substitution rates higher than those of vertebrates have been observed also for the larvacean *Oikopleura dioica* [22, 28, 29]. The disparity in rates between ascidians (or more generally tunicates) and vertebrates can either be the result of an acceleration in the former or a slowing down in the latter. The second possibility has been proposed as the most probable by Peterson et al., who compared vertebrate with the remaining metazoan [27]. One may wonder what are the evolutionary forces responsible of the changes in molecular evolutionary pace described above. Changes in mutation rate, population size, selection coefficients, metabolic rate, and generation time have been claimed to affect the variation in substitution rates among species [30, 31]. However, more recent reassessments of these factors did not fully support many of the initial claims. For instance, in spite of the fact that the metabolic rate is approximately one order of magnitude higher in endothermic amniotes than in ectothermic ones of similar body mass [32], the substitution rate was found to be of the same order of magnitude [27]. Moreover, the so-called “generation time effect” was not supported in many cases, since, for instance, rodents and carnivores exhibit close substitution rates [33]. Other analysis and interpretations have been put forward to explain why Ciona evolves faster than vertebrates. In this regard, it is worth mentioning that according to Peterson et al. (2004) [27] the main factor that underlies the different evolutionary rate in chordates is related to the issue of genome duplication. Indeed, two rounds of whole genome duplications took place in vertebrates, the first one before the branching of agnates from the remaining vertebrates, but after the separation of ascidians, and the second one just before the split of cartilaginous fish and bony vertebrates [29]. According to this point of view, vertebrate genomes would evolve more slowly as a result of an increased number of protein-protein interactions which implies that a higher proportion of surface amino acids would be involved in these interactions. Participating in specific protein-protein interactions limits the possibility to vary; namely, the participant amino acids would be under more stringent selective constrains [34]. This double duplication hypothesis nevertheless is at odds with the classical hypothesis proposed by Ohno according to which genome duplication results in higher evolutionary rate because one of the gene copies would be more free to accept mutations [35]. We can conclude that further data and analysis are required in order to shed light on this controversial point. 

## Supplementary Material

Average distance between C. intestinalis, vertebrate and out group.

## Figures and Tables

**Table 1:** Genomewide average amino acid distances, their standard error, and the time of divergence in million years (My) for each pair of vertebrate species analyzed.

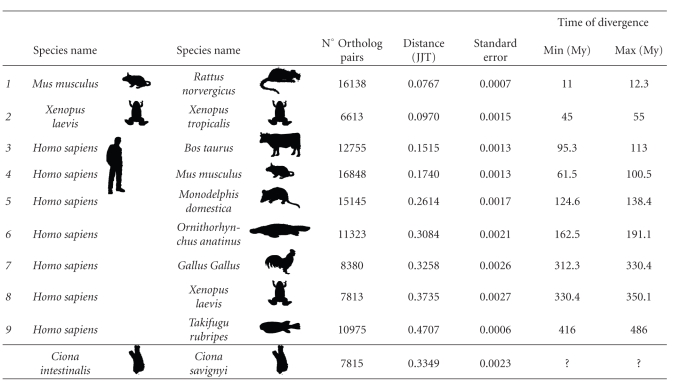

**Table 2:** Tajima's Relative Rate Test, orthologous sequences from (1) *C. intestinalis*, (2) one vertebrate and (3) an outgroup (Amphioxus above, Sea Urchin below). *Related to the total of significant differences.

Species name	N° ortholog trios	D13 > D23 (%)	Total significant	Significant at 1%	Significant at 5%	D13 > D 23 Significant (*)
*2-Homo sapiens*	5911	4603 (77.9%)	3438	2736	702	2998 (87.2%)
*2-Mus musculus*	5862	4519 (77.1%)	3422	2691	731	2949 (86.2%)
*2-Xenopus laevis*	4452	3410 (76.6%)	2587	2012	575	2180 (84.3%)
*2-Ornithorhynchus anatinus*	5392	3605 (66.9%)	2941	2263	678	2158 (73.4%)
*2-Monodelphis domestica*	5928	4468 (75.4%)	3408	2653	755	2904 (85.2%)
*2-Bos taurus*	4864	3906 (80.3%)	3075	2493	582	2704 (87.93)
*2-Gallus gallus*	5221	3975 (76.1%)	3131	2466	665	2640 (84.3%)
*2-Takifugu*	4918	4080 (83.0%)	3064	2464	600	2797 (91.3%)
Total	**42548**	**32566 (76.5%)**	**25066**	**19778**	**5288**	**21330 (85.1%)**
*1-Ciona intestinalis*						
*3-Amphioxus (Branchiostoma floridae)*						

*2-Homo sapiens*	4662	3720 (79.8%)	2485	1873	612	2232 (89.8%)
*2-Mus musculus*	4659	3659 (78.5%)	2449	1880	569	2149 (87.8%)
*2-Xenopus laevis*	3670	2847 (77.6%)	1936	1432	504	1683 (86.9%)
*2-Ornithorhynchus anatinus*	4093	2322 (56.7%)	2063	1563	500	1363 (66.1%)
*2-Monodelphis domestica*	4623	3570 (77.2%)	2407	1812	595	2076 (86.2%)
*2-Bos taurus*	4431	3400 (76.3%)	2321	1714	607	1985 (85.5%)
*2-Gallus gallus*	4166	3261 (78.3%)	2243	1703	540	1936 (86.3%)
*2-Takifugu*	4457	3529 (79.2%)	2310	1716	594	2048 (88.7%)
Total	**34761**	**26308 (75.7%)**	**18214**	**13693**	**4521**	**15472 (84.9%)**
*1-Ciona intestinalis*						
*3-Sea urchin (Strongylocentrotus purpuratus)*						

**Table 3:** *Substitution rates*. Average distance of orthologous sequences from (1) *C*. *intestinalis*, (2) one vertebrate, and (3) *B*. *floridae*. a - correspond to the distance between *C. intestinalis* and the common ancestor with vertebrate, and b is the distance between vertebrate and the common ancestor with *C. intestinalis*. See Figure  1S, in supplementary materials.

	d12 JTT	d13 JTT	d23 JTT	**a**	**b**	(a-b)/b
*2-Homo sapiens*	0.6590	0.6501	0.5138	0.3976	0.2613	0.5217
*2-Mus musculus*	0.6608	0.6472	0.5195	0.3942	0.2666	0.4789
*2-Xenopus laevis*	0.6377	0.6316	0.4917	0.3888	0.2489	0.5621
*2-Bos taurus*	0.6638	0.6471	0.5193	0.3958	0.2680	0.4769
*2-Gallus gallus*	0.6517	0.6439	0.5082	0.3937	0.2580	0.5258
*2-Takifugu rubripes*	0.6684	0.6497	0.5273	0.3954	0.2730	0.4486

1-*Ciona intestinalis *					*Average*: **0.5023**	
3-Amphioxus* (Branchiostoma floridae) *						
