# Supplementary figures and images for: How Fast Is the Sessile Ciona?
Source: Comp Funct Genomics. 2009 Dec 16;2009:875901. doi: 10.1155/2009/875901 (PMC2801007; doi:10.1155/2009/875901)

Figure 1S

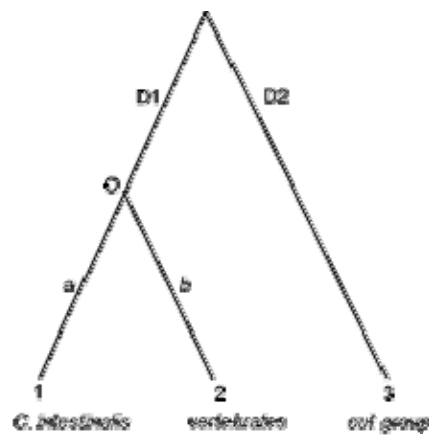

Supplement: Supplementary file 1 — Average distance between C. intestinalis, vertebrate and out group. [file 875901.f1.pdf]
